# Screening of lactic acid bacteria with anti-adipogenic effect and potential probiotic properties from grains

**DOI:** 10.1038/s41598-023-36961-0

**Published:** 2023-07-07

**Authors:** Min Ju Seo, Sung-Min Won, Min Ju Kwon, Ji Hyeon Song, Eun Bee Lee, Jun Hyeong Cho, Kye Won Park, Jung-Hoon Yoon

**Affiliations:** Department of Food Science and Biotechnology, Sungkyunkwan University, Jangan-gu, Suwon, Republic of Korea

**Keywords:** Biotechnology, Microbiology, Health care

## Abstract

A total of 187 lactic acid bacteria were isolated from four types of grains collected in South Korea. The bacterial strains were assigned as members of *Levilactobacillus brevis, Latilactobacillus curvatus, Lactiplantibacillus plantarum*, *Lactococcus taiwanensis*, *Pediococcus pentosaceus*, and *Weissella paramesenteroides* based on the closest similarity using 16S rRNA gene sequence analysis. The strains belonging to the same species were analyzed using RAPD-PCR, and one or two among strains showing the same band pattern were selected. Finally, 25 representative strains were selected for further functional study. Inhibitory effects of lipid accumulation were observed in the strains tested. *Pediococcus pentosaceus* K28, *Levilactobacillus brevis* RP21 and *Lactiplantibacillus plantarum* RP12 significantly reduced lipid accumulation and did not show cytotoxicity in C3H10T1/2 cells at treatment of 1–200 μg/mL. The three LAB strains decreased significantly expression of six adipogenic marker genes, *PPARγ*, *C/EBPα*, *CD36*, *LPL*, *FAS* and *ACC*, in C3H10T1/2 adipocytes. The three strains survived under strong acidity and bile salt conditions. The three strains showed adhesion to Caco-2 cells similar to a reference strain LGG. The resistance of the three strains to several antibiotics was also assessed. Strains RP12 and K28 were confirmed not to produce harmful enzymes based on API ZYM kit results. Based on these results, strains K28, RP21 and RP12 isolated from grains had the ability to inhibit adipogenesis in adipocytes and potentially be useful as probiotics.

## Introduction

Diet-induced obesity is a major factor in many chronic diseases such as cardiovascular disease, non-alcoholic fatty liver disease (NAFLD) and type2 diabetes, and is considered a serious health concern^1,2^. Currently, drug treatment and surgery with mechanisms that inhibit lipid absorption and suppress appetite are used worldwide to prevent and treat obesity. However, in the case of drugs, continuous administration is limited due to side effects, and a risk of complications in surgery is possible^3,4^. Therefore, non-drug therapies that are safer and improve the balance of metabolism are needed to treat and prevent obesity and probiotics have been proposed as an alternative^5^. Probiotics are defined as live microorganisms that provide the host with health benefits when administered in proper quantities^6^. It has been shown in many studies that the probiotics are effective in alleviation of intestinal inflammation, allergy prevention, reduced total serum cholesterol and inflammation and anti-cancer activity^7–10^. Among them, several probiotics are also known to have potential anti-obesity effects such as reduced body fat, blood sugar control and cholesterol improvement^11,12^. Lactic acid bacteria (LAB) is the main bacteria in probiotics^13^. According to the American Food and Drug Administration, LAB are generally regarded as safe (GRAS). LAB is widely used in the food industry, and studies of LAB functionality are growing in popularity. In several recent studies, LAB was shown to alleviate obesity by decreasing body weight, improving inflammatory state or glucose tolerance, and altering gut microbiota in diet-induced obese mice^14–16^.

Grains are a major staple food in Asia. Grains mainly contain carbohydrates including fiber and oligosaccharides, which makes them an excellent source of prebiotics defined as “a nondigestible food ingredient that beneficially affects the host by selectively stimulating the growth and/or activity of one or a limited number of bacteria in the colon, and thus improves host health”^17,18^. A microbial community composed of LAB was shown to exist in grains^18^. In a previous study, LAB isolated from grains exerted antimicrobial properties^19^; however, few studies about useful properties, including anti-obesity effects, have been performed.

The objectives of the present study were to isolate LAB from various grains collected in the Republic of Korea and to screen lactic acid bacteria with inhibition of adipogenesis. The selected strains with anti-adipogenic effects were investigated to determine whether they have useful properties as probiotic candidates for development as functional food.

## Material and methods

### Isolation of LAB strains and growth conditions

Four types of grains, rice, brown rice, black rice and hulled barley, were collected in the Republic of Korea. The grains were ground using blender and enriched for 7 days with distilled water under aerobic conditions. The grain samples were serially diluted with 0.85% (w/v) saline solution and spread on De Man-Rogosa-Sharpe (MRS; BD Difco, Sparks, MD, USA) agar. After incubation for 48 h at 30 ℃ or 37 ℃, LAB were isolated from the MRS agar plates and cultivated aerobically for 18 h at 30 ℃ or 37 °C in MRS agar. *Lacticaseibacillus rhamnosus* LGG (KCTC 5033), which was used as the experimental control strain for comparative analyses, was purchased from the Korean Collection for Type Cultures (Daejeon, Republic of Korea), and cultured at 37 ℃. All strains, including the experimental control strain, were stored at − 80 ℃ after suspension in 20% (w/v) glycerol solution (Georgiachem, GA, USA).

### 16S rRNA gene sequence analysis and random amplified polymorphic DNA-polymerase chain reaction (RAPD-PCR) analysis

Genomic DNA was extracted using a G-spin genomic extraction kit (iNtRON, Seongnam, Republic of Korea), according to the manufacturer’s protocol. Polymerase chain reaction (PCR) amplification, purification, and sequencing of 16S rRNA gene were performed as described previously^20^. Identification of the closest phylogenetic species based on 16S rRNA gene sequence was performed using the EzBioCloud server (https://www.ezbiocloud.net/)^21^.

Random amplified polymorphic DNA-PCR (RAPD-PCR), which was used to exclude replicates among LAB strains, was performed using two primers, ERIC2 (5′-AAGTAAGTGACTGGGGTGAGCG-3′) and ERIC1R (5′-ATGTAAGCTCCTGGGGATTCAC-3′) as described previously^22^. The PCR products were electrophoresed on 1.5% (w/v) agarose (LPS Solution, Daejeon, Republic of Korea) gel for 60 min, and after electrophoresis, the gel was stained with RedSafe (iNtRON, Seongnam, Republic of Korea).

### Preparation of cell extract from LAB

Cell extracts of LAB were prepared as described previously^23^ with minor modifications. The cell mass was harvested using centrifugation and washed twice with phosphate-buffered saline (PBS, pH 7.2). The washed cells were resuspended in distilled water at a concentration of 100 mg/mL and sonicated using the method described previously^16^. The sonicated cell extracts were centrifuged at 13,000 rpm for 15 min at 4 °C and the supernatants were filtered using a 0.45 µM syringe filter (Sartorius Stedim Biotech GmbH, Göttingen, Germany) and lyophilized. The resulting powder was dissolved in sterile water to appropriate concentrations.

### Cell culture, adipocyte differentiation and intracellular triglyceride content

C3H10T1/2 cells were purchased from the American Type Culture Collection (Manassas, VA, USA) and cultured following the method described previously^24^. A subculture of C3H10T1/2 cells was performed with Dulbecco's modified Eagle's medium (DMEM) containing 10% fetal bovine serum (FBS; Hyclone, Logan, UT, USA) and antibiotics (penicillin and streptomycin, Hyclone). After seeding in 12-well plates, C3H10T1/2 cells were cultured in DMEM containing 10% FBS and antibiotics until confluency. Confluent cells were induced into adipocytes in DMEM supplemented with 10% FBS, antibiotics, 20 nM GW1929 (Sigma), 0.5 mM 3-isobutyl-1-methylxanthine (Sigma-Aldrich, St. Louis, MO, USA), 1 μM dexamethasone (Sigma-Aldrich), and 10 μg/mL insulin (Sigma-Aldrich). After 48 h, the differentiating cells were refreshed with media containing DMEM, 10% FBS, 20 nM GW1929, and 10 μg/mL insulin.

Cell extracts of LAB were adjusted by suspending to a concentration of 25, 50, and100 μg/mL with sterile distilled water to create the same conditions. LAB cell extracts were treated during adipocyte differentiation of C3H10T1/2 cells, and sterile distilled water was treated as control. Then, the differentiated C3H10T1/2 cells were fixed with 4% formaldehyde (Sigma-Aldrich) in PBS (Hyclone) at room temperature overnight and stained with Oil Red O (Sigma-Aldrich). To quantify intracellular triglyceride content, stained cells from at least two independent experiments were resolved in isopropanol (Sigma-Aldrich) and measured with a spectrophotometer at 520 nm.

### Cell viability assay

Cell viability was determined using methyl thiazolyl tetrazolium salt (MTT) colorimetric assays (ab197010, Abcam). C3H10T1/2 cells were seeded at 1.5 × 10^4^ cells per well in 96-well plates, then treated with various strain doses (1, 12.5, 25, 50, 100, and 200 μg/mL) and sterile distilled water as control in triplicate. After 24 h, MTT (20 μL/100 μL in medium) was added into the media and cells incubated for 4 h at 37 ℃. The absorbance of formazan dye was measured at 490 nm using a microplate reader (BioTek, Winooski, VT, USA).

### Quantitative real-time polymerase chain reaction (RT PCR) analysis

Total RNA was extracted from C3H10T1/2 cells using QIAzol lysis reagent (QIAGEN, Germantown, MD, USA). First-strand complementary DNA was synthesized from 0.5 μg of total RNA using ReverTra Ace Master Mix (Toyobo, Osaka, Japan) according to the manufacturer’s instructions. Quantitative RT PCR was performed in 25 μL final reaction volume containing Power SYBR Premix ExTaq (RP041A; Takara, Shiga, Japan), primers, and cDNA using thermal cycler machine (Takara). The primer sequences used for the PCR were described previously^24^.

### Tolerance assays against acid and bile salts

Tolerance to acid was measured as described previously^25^ with minor modifications. LAB strains were cultured overnight (18 h) at 30 °C (for RP21 and K28) or 37 °C (for RP12 and LGG), harvested for 10 min at 7000 rpm at 4 °C, and washed twice with PBS buffer (pH 7.2). Bacterial cells (approximately 10^9^ CFU/mL) were resuspended in liquid MRS medium (pre-adjusted to pH 1.0, 2.0, 2.5, and 3.0) and incubated for 3 h at 30 or 37 °C. Viability was determined in triplicate in terms of viable colony counts using the plate count method. Tolerance to bile salts was measured as described previously^26^ with minor modifications. LAB strains were suspended in liquid MRS medium containing 0.3, 0.5, 1.0, and 2.0% oxgall (Sigma-Aldrich) at a concentration of approximately 10^9^ CFU/mL. After incubation for 6 h at 30 °C or 37 °C, suspension was poured into MRS agar plates and incubated at optimum growth temperatures for 48 h. Tolerance assays against acid and bile salts were performed in triplicate and LGG was used as the comparative strain.

### In vitro adhesion assays

The adherence assay was performed according to the method described previously^27^ with minor modifications. Caco-2 cells used for the adherence assay were purchased from the Korean Cell Line Bank (Seoul, Korea). The Caco-2 cells were cultured in high glucose DMEM supplemented with 10% (v/v) FBS (Hyclone) and 1% (v/v) penicillin–streptomycin at 37 °C in 5% CO_2_ atmosphere. The Caco-2 cells were seeded at 2 × 10^5^ cells/well in 6-well tissue culture plates. The adherence assay was performed at post-confluence. The monolayer was washed with sterile PBS (Hyclone) twice. LAB cells were diluted with DMEM to approximately 10^9^ CFU/mL and added to the wells. Plates were incubated for 90 min at 37 °C in 5% CO_2_ atmosphere. The Caco-2 monolayers were washed three times with sterile PBS (Hyclone) and treated with EDTA-trypsin solution for 3 min. The cell suspensions were serially diluted and spread on MRS agar plates. Cell viability was counted after incubation for 48 h. The adhesion ability of LAB was calculated as the percentage between remaining bacteria and initial bacteria per well. The same passage Caco-2 cells were used in adhesion assays and assays were repeated in triplicate.

### Antibiotic susceptibility

Susceptibility to antibiotics was examined using the disc-diffusion method with application of modified agar diffusion method described previously^28,29^. LAB inoculated in MRS agar were adjusted to approximately 10^8^ CFU/mL and paper discs (Advantec, Tokyo, Japan) were dispensed. Each disc was treated with 10 μL of specific antibiotic. The concentrations of antibiotics tested are listed in Supplementary Table 1. The inhibition zone diameters were measured and evaluated in terms of sensitive, intermediate sensitive, and resistant according to the interpretative standard table (Supplementary Table 1). The 2013 Clinical and Laboratory Standards Institute criteria^30^ were used for interpretation.

### Enzyme activity test

Enzyme activity of the LAB was investigated as described previously^16^ using the API ZYM kit (BioMérieux, Marcy l’Etoile, France).

### Statistical analysis

Results are presented as mean ± standard error of the mean (SEM) of three independent experiments. Significance differences between groups in triglyceride content were determined using Duncan's multi-range test. Significance differences in gene expression and adhesion ability were determined by comparison with control using two-tailed unpaired Student’s *t*-test. A p-value < 0.05 was considered statistically significant. Statistical analyses were performed using SPSS Inc. software (version 19.0).

## Results and discussion

### Isolation and identification of LAB strains from grains

Bacterial strains were isolated from four types of grains collected in the Republic of Korea and a total of 187 LAB strains were obtained through 16S rRNA gene sequencing followed by identification. From the 16S rRNA gene sequence analyses of the LAB strains, 20 strains had the closest similarities to the type strain of *Levilactobacillus* (previously *Lactobacillus*) *brevis*, 3 strains had the closest similarities to the type strain of *Latilactobacillus* (previously *Lactobacillus*) *curvatus*, 22 strains had the closest similarities to the type strain of *Lactiplantibacillus* (previously *Lactobacillus*) *plantarum*, 17 strains had the closest similarities to the type strain of *Lactococcus taiwanensis*, 82 strains had the closest similarities to the type strain of *Pediococcus pentosaceus*, and 43 strains had the closest similarities to the type strain of *Weissella paramesenteroides* (Table 1). The genus *Lactobacillus* has been recently reclassified as 25 genera including *Levilactobacillus*, *Latilactobacillus*, and *Lactiplantibacillus*^31^. *Levilactobacillus brevis, Latilactobacillus curvatus, Lactiplantibacillus plantarum, Pediococcus pentosaceus*, and *Weissella paramesenteroides* have been shown to be isolated from grains^32,33^.

**Table 1:** Lactic acid bacteria (n = 25) isolated from the four kinds of grains and a reference strain (LGG) used in this study.

Selected strain	Closest species by 16S rRNA gene sequence analysis	16S rRNA gene sequence similarity (%)	Isolation source	Number of strain selected
RP20, RP21	*Levilactobacillus brevis*	99.93	Rice	2
RP42, RP50	*Latilactobacillus curvatus*	100	Rice	2
RP11, RP12	*Lactiplantibacillus plantarum*	100	Rice	2
B5, B6	*Lactococcus taiwanensis*	99.93	Black rice	2
H2, H6	*Pediococcus pentosaceus*	100	Brown rice	2
K21, K22		100	Hulled barley	2
H13, H15		100	Brown rice	2
K28		100	Hulled barley	1
H11		100	Brown rice	1
K25		100	Hulled barley	1
B12, B13	*Weissella paramesenteroides*	99.93	Black rice	2
B32, B45		99.93	Black rice	2
H8, H23		99.93	Brown rice	2
B40, H7		99.93	Brown rice, Black rice	2
LGG (KCTC 5033)	*Lacticaseibacillus rhamnosus*	100	Reference strain	1

In RAPD-PCR analysis, six different band patterns were assigned to 82 strains with the closest 16S rRNA gene sequence similarities to *Pediococcus pentosaceus*, and four different band patterns were assigned to 43 strains with the closest 16S rRNA gene sequence similarities to *Weissella paramesenteroides* (Supplementary Fig. 1). The strains assigned as *Levilactobacillus brevis, Latilactobacillus curvatus, Lactiplantibacillus plantarum*, and *Lactococcus taiwanensis* each showed only one type of band pattern (Supplementary Fig. 1). Finally, two representative strains from each group, except for the three groups having only one strain, were randomly selected, and used for further functional characterization (Supplementary Fig. 1; Table 1).

### Screening of strains with anti-adipogenic effects

**Figure 1:**
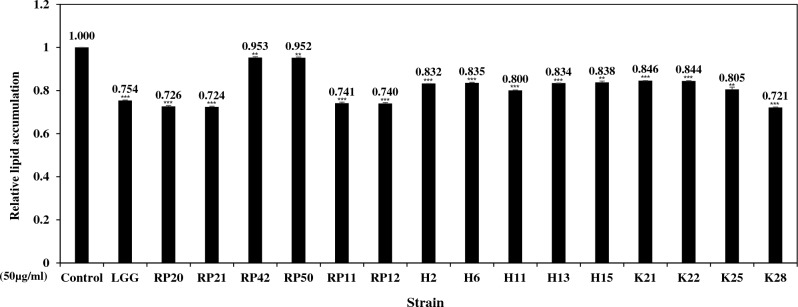
Inhibitory effect on lipid accumulation of LAB strains isolated from grains. Each strain was treated as cell extract at a concentration of 50 μg/mL. Values of each sample were determined relative to control. Values are presented as the mean ± SEM of three independent experiments. Significant differences between selected strains and control are indicated as **p < 0.01, and ***p < 0.001.

Inhibitory effects of lipid accumulation were tested by treating the LAB cell extract on C3H10T1/2 cells. Because the strains assigned to *Lactococcus taiwanensis* and *Weissella paramesenteroides* caused cell damage during the treatment process, they were excluded from the test. A wide range of inhibitory effects of lipid accumulation was observed in the strains selected. Among the strains tested, five strains (*Pediococcus pentosaceus* K28; *Levilactobacillus brevis* RP20 and RP21; *Lactiplantibacillus plantarum* RP11 and RP12) reduced lipid accumulation by more than 20% compared with the control, indicating that they have anti-adipogenic effects (Fig. 1). The two strains (RP20 and RP21) assigned to *Levilactobacillus brevis* and the two strains (RP11 and RP12) of *Lactiplantibacillus plantarum* showed similar results, respectively (Fig. 1). Thus, one strain from RP20 and RP21 and one strain from RP11 and RP12 were selected, and the three strains (K28, RP21 and RP12) were used for further experiments. The above results indicate that the components of LAB cell extract might influence the adipocyte differentiation process, thereby suppressing fat production.

Significant diversity exists among LAB strains regarding functional characteristics that benefit health, such as antioxidant, antitumor, immunomodulatory, and hypocholesterolemic activities^34–40^. In several studies, the cellular components of LAB have shown beneficial effects on improving health^38,41,42^. It is not clear which substance(s) in LAB cell extract induce the anti-adipogenic effects. Exopolysaccharide (EPS) has been known to have anti-adipogenic effects^23^. The EPS, a cell wall component of LAB cells, is loosely associated with the cell envelope and easily released into the surrounding environment^43,44^.

### Effects of LAB strains on cell viability of C3H10T1/2

**Figure 2:**
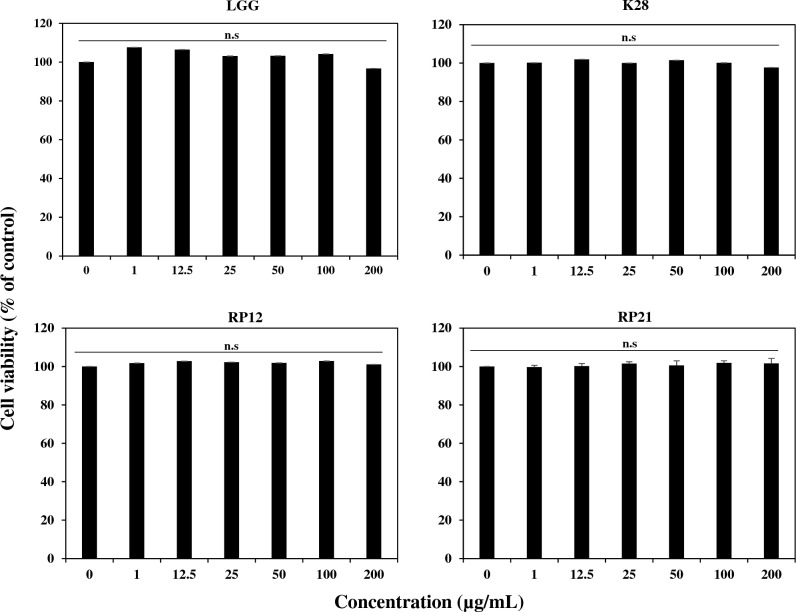
Effects of LAB treatment at different concentrations on viability of C3H10T1/2 cells. The C3H10T1/2 cells were treated with sterile distilled water (control) or LAB strains and their viability was determined using the MTT assay. Data are presented as the mean ± SEM from three independent experiments.

The cytotoxicity at various concentrations of strains LGG, K28, RP21, and RP12 on C3H10T1/2 cells was investigated by measuring cell viability using the MTT assay. C3H10T1/2 cells were found viable at all treatment concentrations of the four strains (Fig. 2). These results indicate that strains LGG, K28, RP21, and RP12 cause no damage to C3H10T1/2 cells^45^.

### Inhibition of adipogenic gene expression by LAB extract during adipocyte differentiation

**Figure 3:**
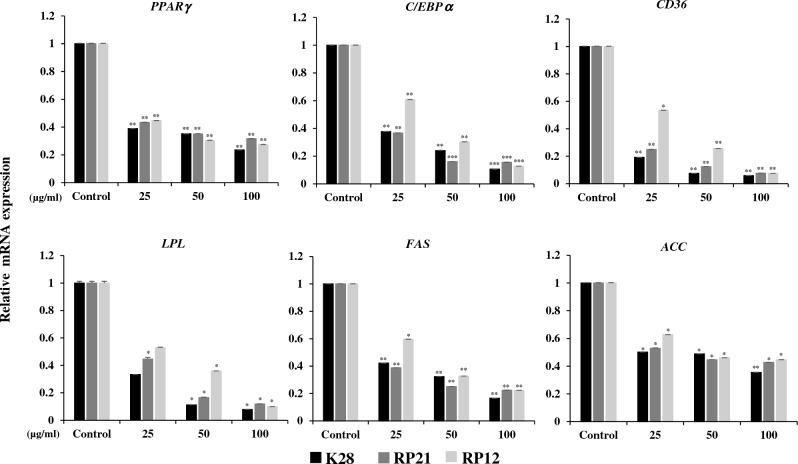
Effects of selected LAB treatment on expression of adipogenic genes in C3H10T1/2 cells. The C3H10T1/2 cells were treated with the indicated concentrations during differentiation for 6 days and expression of adipocyte markers was measured. The mRNA expression levels of peroxisome proliferator-activated receptor γ (*PPARγ*), CCAAT-enhancer-binding protein-α (*C/EBPα*), lipoprotein lipase (*LPL*), fatty acid synthase (*FAS*), cluster of differentiation 36 (*CD36*), and acetyl-coenzyme A carboxylase (*ACC*) were measured using quantitative real-time polymerase chain reaction. Data are expressed as mean ± SEM of three independent experiments. Significant differences between selected strains (*Pediococcus pentosaceus* K28, *Levilactobacillus brevis* RP21 and *Lactiplantibacillus plantarum* RP12) and control are indicated as *p < 0.05, **p < 0.01, and ***p < 0.001.

Inhibition of adipogenesis by strains K28, RP21 and RP12 was investigated by measuring expression of six adipogenic genes using quantitative RT PCR (Fig. 3). *PPARγ* and *C/EBPα* are transcription factors that regulate the process of adipocyte differentiation^46,47^. In addition, the activation of *PPARγ* promotes the expression of adipogenic genes, such as *CD36* and *LPL*, which are important for the uptake and storage of triglycerides^48^. The down regulation of these adipogenic genes may affect decreased lipid accumulation in cells. Fatty acid synthase (*FAS*) gene is a downstream adipocyte gene that contributes to fatty acid synthesis^49^. Acetyl-coenzyme A carboxylase (*ACC*) is another key enzyme for fatty acid synthesis that catalyzes the synthesis of malonyl-CoA^50^.

Cell extracts from strains K28, RP21, and RP12 decreased the expression of adipocyte-related genes in C3H10T1/2 cells (Fig. 3). The expression of the six genes decreased proportionally with increasing concentrations of the extracts of the three strains (Fig. 3). The three strains significantly reduced (p < 0.01 or 0.001) the expression of *PPARγ* and *C/EBPα* in all concentrations tested. In addition, expression of four other genes associated with adipogenesis, was significantly reduced (p < 0.05 or 0.01) in the three strains, except for *LPL* expression in 25 μg/mL treatment of strains K28 and RP12. Strain K28, which showed the lowest lipid accumulation, was analyzed to have the lowest values in expressions of the six genes after 100 μg/mL treatment (Fig. 3). These results indicate that the three strains may have anti-adipogenic effects by inhibiting the expression of adipogenesis-related genes.

### Tolerance against acid and bile salts

To have specific functionality, a probiotic must reach the intestines alive with resistance to acid and bile salts^51^. The acid tolerance of the selected strains and LGG as a reference strain was examined after incubation for 3 h in pH 3.0, 2.5, 2.0, and 1.0 (Table 2). The three strains and LGG maintained the values of more than 9 log CFU/mL at pH 3. Under pH 2.5 condition, the survival rates of strains K28 and RP21 decreased more than 2 log and approximately 1 log, respectively, whereas strains RP12 and LGG showed decreases of more than 3 log and 2 log, respectively (Table 2). Under pH 2 condition, the survival rate of strains K28 and RP21 decreased approximately 3 log and 2 log, respectively, and the survival rate of strains RP12 and LGG decreased approximately 6 log (Table 2). The three strains, except RP21 with approximately 3 log CFU/mL, showed low viability of less than 2 log CFU/mL at pH 1 (Table 2). Strain RP21 was also found to have higher acid resistance, as a strain of *Levilactobacillus* (*Lactobacillus*) *brevis* was shown highly acid-resistant in a previous study^52^. The pH of gastric fluid in the body is maintained at approximately 3.0, and probiotics are generally known to be highly acid-resistant if they are maintained at pH 3 for approximately 3 h^53^. Thus, the three strains were concluded to be highly tolerant to acid. Because food matrix can help the survival of LAB in the gastrointestinal tract due to its buffering capacity, the strains are expected to have stronger viability when used with carrier foods^54^.

**Table 2:** Acid and bile tolerance of strains K28, RP21 and RP12 and a reference strain (LGG) (log CFU/ml).

Strain	Acid condition										Bile salts								
	pH 3.0		pH 2.5				pH 2.0				pH 1.0				0.3%		0.5%	1.0%	2.0%
	Initial mean counts	3 h	Initial mean counts		3 h		Initial mean counts		3 h		Initial mean counts		3 h						
K28	9.58 ± 0.08	9.56 ± 0.04	9.63 ± 0.01		7.30 ± 0.05***		9.74 ± 0.01		6.76 ± 0.01***		9.82 ± 0.01		1.78 ± 0.04***		+ ^a^		+	+	+
RP21	9.71 ± 0.07	9.59 ± 0.03	9.87 ± 0.01		8.66 ± 0.01**		9.73 ± 0.01		7.65 ± 0.01***		9.96 ± 0.04		3.41 ± 0.06***		+		+	+	+
RP12	9.20 ± 0.04	9.18 ± 0.05	9.24 ± 0.01		5.38 ± 0.09***		9.24 ± 0.01		3.15 ± 0.10***		9.12 ± 0.04		1.65 ± 0.09***		+		+	+	+
LGG	9.55 ± 0.03	9.59 ± 0.09	9.34 ± 0.04		6.94 ± 0.03***		9.48 ± 0.07		3.56 ± 0.09***		9.58 ± 0.09		1.76 ± 0.03***		+		+	+	+

All values are mean ± SEM (n = 3).

Significant differences are indicated as ** p < 0.01, and *** p < 0.001.

^a^+, survival.

Bile salts are another factor that can reduce bacterial survival in the gastrointestinal tract by destroying cell membranes^51^. Strains K28, RP21 and RP12 were found to survive after 6 h exposure to 0.3, 0.5, 1.0, and 2.0% bile salts, similar to LGG which is known to be highly resistant to bile salts (Table 2). Although the in vitro assay cannot provide the same conditions as the gastrointestinal tract, it is recognized as an effective evaluation method to select potential strains when using proper criteria^27^.

### Adherence to Caco-2 cells

**Figure 4:**
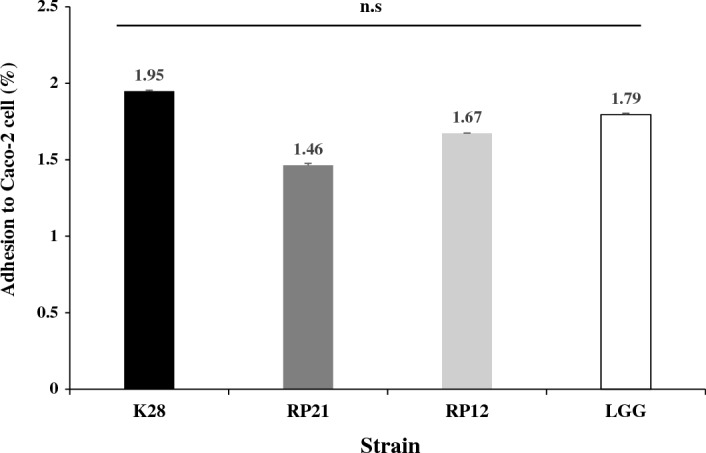
Adhesion ability of *Pediococcus pentosaceus* K28, *Levilactobacillus brevis* RP21, and *Lactiplantibacillus plantarum* RP12 to Caco-2 epithelial cells compared with the reference strain LGG. Data are expressed as mean ± SEM of three independent experiments.

The adhesion ability of probiotics is a main factor that can increase the possibility of their survival and colonization in the gastrointestinal tract^55^. Adhesion is also required to prevent attachment of pathogenic bacteria through competition in intestinal epithelium^56^. Thus, the adherence ability has been considered an important biological property for the selection of useful probiotic strains^57^. In the present study, the adhesion ability of the three strains was evaluated using Caco-2 cells, which have morphological and physiological properties of human enterocytes, and their adhesion abilities were compared with that of the reference strain LGG (Fig. 4). Strain K28 had stronger adhesion ability than those of LGG and the two other strains (Fig. 4). The adhesion ability of strain K28 was highest at 1.95%, followed by LGG (1.79%), RP12 (1.67%), and RP21 (1.46%).

### Antibiotic susceptibility

Probiotics have been widely used in various fields including food and medical industries. Antimicrobial sensitivity for evaluation of probiotics is considered important for safety, because the resistant genes can be horizontally transferred to pathogenic bacteria, which can become a serious threat^58^. Sensitivity results of the strains for nine antibiotics used in this study are listed in Supplementary Table 1. For the nine antibiotics tested, strains K28, RP21 and RP12 showed sensitivity patterns similar to strain LGG. In this study, all four strains were equally sensitive to chloramphenicol and rifampicin, whereas strains K28 and RP21 were intermediate sensitive to tetracycline and strains RP12 and LGG were sensitive to tetracycline (Supplementary Table 1). Sensitivity or intermediate sensitivity of *Lactobacillus* species and *Pediococcus* species to chloramphenicol and tetracycline has been previously reported^59,60^. Strains K28, RP21, RP12 and LGG were resistant to gentamycin, kanamycin, and streptomycin, which are known to inhibit protein synthesis targeting Gram-negative bacteria. The resistance to aminoglycoside antibiotics is an intrinsic property among *Lactobacillus* species and *Pediococcus* species^61^. Therefore, the three strains are unlikely to cause safety problems based on antibiotic susceptibility profile tested.

### Enzyme production

For the safety of probiotic strains, it may be required to assess whether the strains produce harmful enzyme. β-glucuronidase is known as the carcinogen enzyme, which may increase the likelihood of tumor induction in the colon^62,63^. When the three strains were evaluated using API ZYM kit, strains RP12 and K28 did not produce any harmful enzymes such as β-glucuronidase, but strain RP21 was observed to produce β-glucuronidase (Supplementary Table 2).

## Conclusion

In the present study, 187 LAB strains were isolated from four types of grains and identified using 16S rRNA gene sequence analysis. The 25 strains selected based on RAPD-PCR analysis were subjected to functional characterization. Among the strains tested, *Pediococcus pentosaceus* K28, *Levilactobacillus brevis* RP21, and *Lactiplantibacillus plantarum* RP12 had the potential to be useful probiotic candidates based on several characteristic analyses. The three strains exerted inhibitory effects on lipid accumulation and adipocyte differentiation by decreasing the expression of adipocyte-related genes. In addition, the three strains showed good tolerance against acid and bile salts, good intestinal cell adhesion, and were sensitive to chloramphenicol and rifampicin. In particular, strains RP12 and K28 did not produce β-glucuronidase. Therefore, *Pediococcus pentosaceus* K28, *Levilactobacillus brevis* RP21 and *Lactiplantibacillus plantarum* RP12 were concluded to have potential as probiotic candidates for use as functional neutraceutical foods.

## Supplementary Information

Supplementary Information.

## Data Availability

16S rRNA gene sequences of strains K28, RP21 and RP12 have been deposited in the National Centre for Biotechnology Information (NCBI) under GenBank accession numbers ON724233, ON724232 and ON724263, respectively.
