# Supplementary material for: Screening of lactic acid bacteria with anti-adipogenic effect and potential probiotic properties from grains
Source: Sci Rep. 2023 Jul 7;13:11022. doi: 10.1038/s41598-023-36961-0 (PMC10329024; doi:10.1038/s41598-023-36961-0)
Supplement: Supplementary file 1 — Supplementary Information. [file 41598_2023_36961_MOESM1_ESM.pdf]

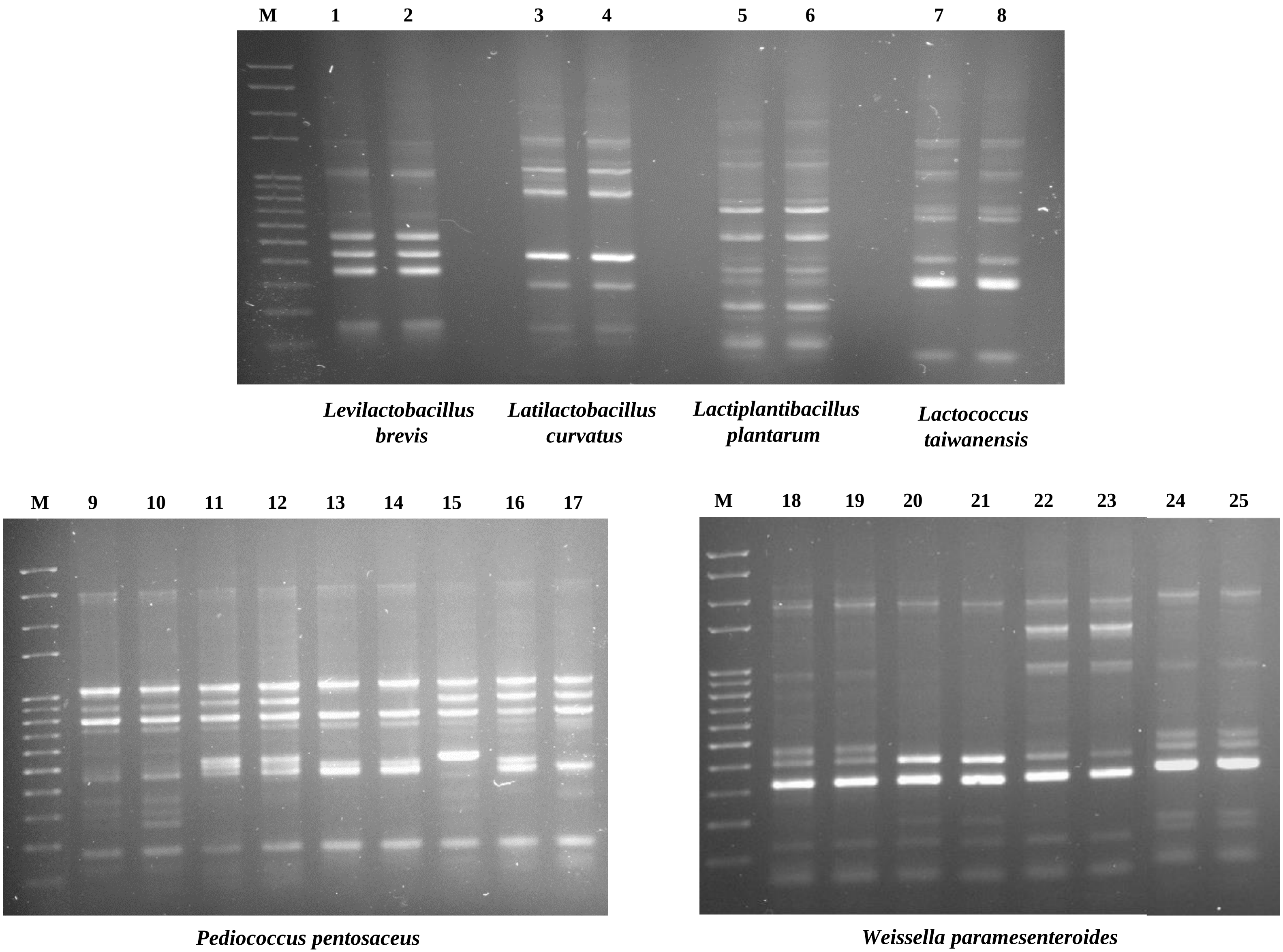


**Supplementary Fig. 1.** RAPD-PCR profiles of 27 Lactic acid bacteria isolated from the four kinds of grains. M, 100-bp DNA ladder (T&I, Chuncheon, Republic of Korea). Strains: 1, RP20; 2, RP21; 3, RP42; 4, RP50; 5, RP11; 6, RP12; 7, B5; 8, B6; 9, H2; 10, H6; 11, K21; 12, K22; 13, H13; 14, H15; 15, K28; 16, H11; 17, K25; 18, B12; 19, B13; 20, B32; 21, B45; 22, H8; 23, H23; 24, B40; 25, H7

**Supplementary Table 1.** Antibiotic susceptibility of strains K28, RP21, RP12 and a reference strain (LGG).

| Antibiotic | Disc conc. (μg) | Interpretative zone diameters (mm)[a] | | | K28 | RP21 | RP12 | LGG |
|---|---|---|---|---|---|---|---|---|
| | | R | I | S | | | | |
| Ampicillin | 10 | ≤12 | 13−15 | ≥16 | R[a] | R | R | R |
| Cephalothin | 30 | ≤14 | 15−17 | ≥18 | S | I | I | I |
| Penicillin G | 30 | ≤13 | 14−17 | ≥18 | R | R | R | R |
| Gentamicin | 10 | ≤12 | 13−14 | ≥15 | R | R | R | R |
| Kanamycin | 30 | ≤13 | 14−17 | ≥18 | R | R | R | R |
| Streptomycin | 10 | ≤19 | 20−27 | ≥28 | R | R | R | R |
| Chloramphenicol | 10 | ≤11 | 12−14 | ≥15 | S | S | S | S |
| Tetracycline | 30 | ≤14 | 15−18 | ≥19 | I | I | S | S |
| Rifampicin | 5 | ≤14 | 15−17 | ≥18 | S | S | S | S |

[a] The interpretation standard referred to the CLSI criteria [30] and Charteris et al [28].

S: Sensitive, I: Intermediate, R: Resistant.

**Supplementary Table 2.** Enzymatic activity of strains K28, RP21, RP12 and a reference strain (LGG).

| Enzyme | K28 | RP21 | RP12 | LGG |
|---|---|---|---|---|
| Control | – | – | – | – |
| Alkaline phosphatase | – | – | + | + |
| Esterase | + | + | + | + |
| Esterase Lipase | + | + | + | + |
| Lipase | + | + | + | + |
| Leucine arylamidase | + | + | + | + |
| Valine arylamidase | + | + | + | + |
| Cystine arylamidase | + | + | + | + |
| Trypsin | – | – | – | – |
| $\alpha$-Chymotrypsin | – | – | – | – |
| Acid phosphatase | + | + | + | + |
| Naphthol-AS-BI-phosphohydrolase | + | + | + | + |
| $\alpha$-Galactosidase | + | + | – | – |
| $\beta$-Galactosidase | + | + | + | + |
| $\beta$-Glucuronidase | – | + | – | – |
| $\alpha$-Glucosidase | + | + | + | + |
| $\beta$-Glucosidase | + | + | + | + |
| N-Acetyl-$\beta$-glucosaminidase | + | – | + | + |
| $\alpha$-Mannosidase | – | – | – | – |
| $\alpha$-Fucosidase | – | – | – | + |

+, Positive; –, Negative.
